# Complete Genome Sequence of Stenotrophomonas maltophilia Siphophage Salva

**DOI:** 10.1128/MRA.00083-21

**Published:** 2021-03-11

**Authors:** Brandi Jefferson, Guichun Yao, James Clark, Tram Le, Carlos Gonzalez, Mei Liu, Ben Burrowes

**Affiliations:** a Department of Biochemistry and Biophysics, Texas A&M University, College Station, Texas, USA; b Department of Plant Pathology and Microbiology, Texas A&M University, College Station, Texas, USA; c Center for Phage Technology, Texas A&M University, College Station, Texas, USA; Queens College

## Abstract

Stenotrophomonas maltophilia is a Gram-negative pathogen causing severe and often refractory illnesses such as pneumonia and bacteremia. We present the genome of phage Salva, a novel S. maltophilia phage that is not closely related to any phages currently deposited in GenBank. The genome is 60,789 bp, containing 102 putative protein-coding genes.

## ANNOUNCEMENT

Stenotrophomonas maltophilia is an emerging, Gram-negative, pathogenic bacterium that is often multidrug resistant and is found in environments like animal by-products, food waste, and other water sources (1). Infections of S. maltophilia are normally acquired nosocomially, can cause significant life-threatening conditions like pneumonia and bacteremia, and are associated with high mortality rates among certain populations of patients (2). Antibiotic-refractory S. maltophilia infections are difficult to treat, so further studies into alternative treatment options, such as phage therapy, are necessary.

Phage Salva was isolated in 2019 from a wastewater sample collected in Cincinnati, Ohio, by enriching the sample using a multidrug-resistant S. maltophilia clinical isolate obtained from human sputum in Decatur, GA, as the host strain aerobically grown at 30°C in nutrient broth (BD), followed by plaque purification (3, 4). To purify the genomic DNA, the modified Promega Wizard DNA cleanup kit protocol was used (5). An Illumina TruSeq Nano low-throughput kit was used to prepare DNA libraries, which were sequenced on an Illumina MiSeq instrument with paired-end 300-bp reads using 500-cycle v2 chemistry. A total of 788,720 raw reads were visualized using FastQC v0.11.9 (www.bioinformatics.babraham.ac.uk/projects/fastqc) and then manually trimmed using the FastX Toolkit v0.0.14 (http://hannonlab.cshl.edu/fastx_toolkit). The genome was closed by PCR (with 5′-ATCGCATCTTCGTCCTTTCC-3′ and 5′-CTTGTGGCGAAGGTATTCCA-3′ as the primer set) and confirmed to be complete by Sanger sequencing. Structural annotations were completed using GLIMMER v3 and MetaGeneAnnotator v1.0 (6, 7). tRNAs were detected with ARAGORN v2.36 (8). To calculate genome-wide sequence similarity, progressiveMauve v2.4 was used (9). Gene functions were predicted using InterProScan v5.33, BLAST v2.9.0, and TMHMM v2.0 (10–12). The sequence similarity search was set at an E value of <0.001 against the NCBI nonredundant and Swiss-Prot/TrEMBL databases. All annotation tools were used with their default settings and hosted (except HHpred) by the Center for Phage Technology (CPT) (https://cpt.tamu.edu/galaxy-pub) (13–15).

Phage Salva has a 60,789-bp genome with a GC content of 56.4%, in comparison to its host genome, which has a GC content of 66.7% (16). A long terminal repeat region (3,973 bp) is predicted by PhageTerm (17) analysis. Phage Salva has one tRNA and 102 putative protein-coding genes, of which 24 have predicted function. Genes that are normally associated with the lysogenic life cycle were not identified in the Salva genome. Salva was predicted to be a siphovirus based on genomic analysis, which was confirmed visually by transmission electron micrography. Salva contains two predicted tape measure chaperone genes, not linked by a programed transcriptional frameshift as is common in many tailed phages (18, 19). A typical Gram-negative lysis cassette was identified in the Salva genome, consisting of a class I holin, an antiholin, an endopeptidase endolysin, and a bimolecular spanin complex (20). Comparative genomic analysis revealed that Salva is not closely related to any known phages or prophage elements currently deposited in GenBank. The only phage that is related to phage Salva is *Stenotrophomonas* phage vB_SmaS_BUCT548 (GenBank accession number MN937349.1), which shares 74.3% nucleotide identity over 55% coverage (distributed across the Salva genome), as determined by BLASTn.

### Data availability.

The Salva genome is deposited under GenBank accession number MW393850. The associated BioProject, SRA, and BioSample accession numbers are PRJNA222858, SRR11558346, and SAMN14609643, respectively.
